# Annotations

**Published:** 1898-08-13

**Authors:** 


### ANNOTATIONS.

### The Purification of Bad Butter.

It is with a certain feeling of regret that we understand that a new industry is about to be introduced in Ireland—namely, the making of good butter from bad. It has long been thought that, if we could once be sure that there was no margarine about, butter could be judged by its taste. What, then, are we to say to a process by which bad rancid butter fat can be turned again into "fresh" butter? The data on which the process is founded are comparatively simple, and the process itself, although somewhat elaborate, is after all only a rewashing and then a remaking of the butter. What is unpleasant about it is that, in future, freshness and cleanness of taste is to be no security that the butter is the fresh product of fresh cream—no guarantee that from cow to butter-pat all has been clean and free from rottenness. The rancidity of butter is due to the liberation of butyric acid, and other volatile acids and their derivatives, through the action of microbes—in other words, through the operation of decomposition. As is well known, this decomposition mostly takes place in butter which has been badly made, for really well-made butter, from which all the casein and buttermilk has been worked out, will keep for a very long time. In the process which has been lately introduced for the removal of these offensive products of decomposition the butter is melted down with a certain quantity of buttermilk, and stirred until a fine emulsion is obtained. Hot air is then drawn through the melted liquid, by which means a churning action is set up, and while the volatile acids are carried off the solid impurities sink to the bottom and are removed. Then a current of cold air is made to take the place of the hot, and under its influence the butter begins to separate in granules as in the ordinary method of churning. The result is admirable; good butter is made from bad, which is no doubt extremely ingenious. Nevertheless we do not like our old faiths to be so disturbed, and we had far rather feel that our pat of "best fresh" was churned out of new cream than that it was "aërated" out of rancid butter.

### Bad Nauheim.

The first week in August may be said to mark a sort of change in this wonderful centre of heart-cures. The season for the Germans and other foreigners has passed its zenith, and the English one is in full swing; but, inasmuch as the former outnumber the latter by nearly a hundred to one, the actual difference is not as yet very noticeable, but it will every day become more so. Nevertheless, the number of baths has gone down from a daily average of 2,500 in July to an average of 2,200, or fewer, in August. The glare of the July sun has given place to a more bearable temperature, and now and then a breeze stirs the leaves of the trees in the almost too thickly-planted park; and the little town does not seem to simmer in its afternoon heat, as is its wont in the long dog-days of the preceding month. The visitor to these baths, whether lay or medical, will be most forcibly struck by two things—first, the extraordinary rapidity with which, in many cases of heart disease, improvement follows the treatment; and, second, by the fact that no untoward event has yet marred the wonderful record of treatment at the bath-houses. And so we are all here again, the young among us seeking for Hygæia like the Pleiades for their lost sister, but with happier results; and the older among us for some return of youth, in which we are not often wholly disappointed. Professor Schott we look upon as the good genius who has revealed the secret and effects the transformation for us. Long may his recently-acquired honours rest on him, and surely never were honours better deserved. Three years since, in an article in this journal, it was said that the waters of Nauheim issued from the earth in an apparently inexhaustible stream; but, in view of the enormous consumption of water during the season, it might be worth considering how long that statement will hold good. It is a pretty little historical "aside" that Voltaire, in his memorable flight from the Court of Frederick the Great, stopped at Nauheim, and noticed these brine-springs; but how little could this great philosopher have foreseen their enormous importance 150 years later!

### Diet and Suicide.

Dr. Haig is of opinion that suicide may be traced to error in diet, the error being the eating of meat, the drinking of beer and of tea, and the smoking of tobacco. His facts all fall comfortably into their places in support of his hypothesis. Are there not more suicides among men than among women, and do not men consume more meat, more beer, and more tobacco than the women? Again, suicide is more common in England than in Scotland, not apparently because the Scotch are a more canny race, but because the English eat more meat and drink more beer, while the Scotch eat less meat and drink whisky instead of beer. After maintaining that suicide was less common among the Scotch, it was perhaps hardly polite, when addressing a Scotch audience, to go on to say that suicide increased with civilisation. But the fact was explained on the ground of the more injurious diet, that of civilised man being more productive of uric acid and thus of suicide, than that which prevails where civilisation is less advanced. Uric acid is, in fact, at the bottom of all this, and, according to Dr. Haig, the incidence of suicide tallies with the daily, annual, and life fluctuations of uric acid in the blood, being commonest when uric acid is most abundant, namely, in the mornings, in spring and summer, and in childhood and the full prime of life. We have no doubt that errors of diet are responsible for much, and, among other things, for a certain number of suicides; nay, we would go farther, and admit that unsuitable diet, derangement of the proper relation between nutrition and waste, and the consequent loading of the tissues and the blood with abnormal products of metabolism, have much to do with that ill-temper and discontent which leads men to lay their hands violently often upon their neighbours, and sometimes on themselves. All this may be taken for granted, but it is at present far from proven that the peccant material is in all cases the same, and still farther are we from being all agreed that uric acid is the origin of the evil.

